# Imperatorin promotes osteogenesis and suppresses osteoclast by activating AKT/GSK3 β/β‐catenin pathways

**DOI:** 10.1111/jcmm.14915

**Published:** 2019-12-28

**Authors:** De‐Yi Yan, Jiahao Tang, Liang Chen, Bingzhang Wang, Sheji Weng, Zhongjie Xie, Zong‐Yi Wu, Zijian Shen, Bingli Bai, Lei Yang

**Affiliations:** ^1^ Department of Orthopaedic Surgery The Second Affiliated Hospital and Yuying Children's Hospital of Wenzhou Medical University Wenzhou China

**Keywords:** bone metabolism, osteoporosis, Wnt3a

## Abstract

Osteoporosis is caused by disturbance in the dynamic balance of bone remodelling, a physiological process, vital for maintenance of healthy bone tissue in adult humans. In this process, a new bone is formed by osteoblasts and the pre‐existing bone matrix is resorbed by osteoclasts. Imperatorin, a widely available and inexpensive plant extract with antioxidative and apoptotic effects, is reported to treat osteoporosis. However, the underlying mechanism and specific effects on bone metabolism have not been elucidated. In this study, we used rat bone marrow‐derived mesenchymal stem cells and found that imperatorin can activate RUNX2, COL1A1 and osteocalcin by promoting the Ser9 phosphorylation of GSK3β and entry of β‐catenin into the nucleus. Imperatorin also enhanced the production of phospho‐AKT (Ser473), an upstream factor that promotes the Ser9 phosphorylation of GSK3β. We used ipatasertib, a pan‐AKT inhibitor, to inhibit the osteogenic effect of imperatorin, and found that imperatorin promotes osteogenesis *via* AKT/GSK3β/β‐catenin pathway. Next, we used rat bone marrow‐derived monocytes, to check whether imperatorin inhibits osteoclast differentiation *via* AKT/GSK3β/β‐catenin pathway. Further, we removed the bilateral ovaries of rats to establish an osteoporotic model. Intragastric administration of imperatorin promoted osteogenesis and inhibited osteoclast in vivo. Our experiments showed that imperatorin is a potential drug for osteoporosis treatment.

## 1 INTRODUCTION

Osteoporosis is a metabolic disease characterized by reduced bone mass and increased risk of fractures, affecting more than 200 million patients globally.^1^, ^2^ Disturbance in the dynamic balance of bone remodelling is the root cause for osteoporosis. Bone remodelling is a physiological process in which a new bone gets formed by osteoblasts, while the original bone matrix is reabsorbed by osteoclasts. It is a pivotal process to maintain healthy bone tissue in adults, and multiple factors affect and regulate this process.^3^, ^4^ Bisphosphonate, oestrogen and raloxifene, which are commonly used to treat osteoporosis, act by restoring the balance between bone formation and resorption. Although these agents increase the bone mass considerably, they have some limitations and side effects, including thromboembolism and oesophageal stimulation.^5^, ^6^, ^7^ The ultimate goal of an anti‐osteoporosis treatment is to improve the function of osteoblast and inhibit the function of osteoclast, simultaneously, without any adverse reactions. To achieve this, we studied imperatorin, a natural compound as a potential anti‐osteoporosis drug.

Canonical Wnt/β‐catenin pathway plays a key role in the differentiation of bone marrow‐derived mesenchymal stem cells (BMSCs) into osteoblasts.^8^ The entry of β‐catenin into the nucleus initiates the transcription of downstream osteogenesis‐related genes and aids the osteogenic differentiation of the cells.^9^, ^10^, ^11^ When the Wnt pathway is not activated, the cytoplasmic β‐catenin gets destroyed by a complex of casein kinase 1 (CK1) and glycogen synthase kinase 3 (GSK3). This complex leads to ubiquitination of β‐catenin and finally, the proteolytic degradation by phosphorylation of its N‐terminal four serine/threonine residues.^12^ Whereas, the GSK3β is phosphorylated by activated AKT into inactive p‐GSK3β.^13^ Thus, we assume that AKT/GSK3β/β‐catenin pathway affects osteogenesis.

Jiake Xu, Jun Zou et al, by down‐regulating the levels of Ca2 + signal transduction and ROS, inhibiting MAPK/NF‐κ B and its downstream NFATc1 and other osteoclast‐related factors to find drugs to inhibit osteoclast activity in the treatment of osteoporosis. Although these drugs showed inhibitory effect on osteoclast activity, they had no effect on osteogenic activity.^14^, ^15^, ^16^ Simply reducing osteoclast activity may interfere with bone formation, reduce bone transformation and bring side effects such as mandibular osteonecrosis. Studies have shown that the proliferation of early osteoclasts requires the activation of β‐catenin; however, the activated β‐catenin inhibits the differentiation of osteoclasts.^17^ This view is consistent with reports by Modarresi et al, who found that overexpression of β‐catenin by adenoviruses can inhibit the osteoclasts differentiation.^18^ John D. Shaughnessy Jr used human osteoclast progenitor cells isolated from multiple myeloma patients and found that its differentiation was inhibited by Wnt pathway.^19^

Imperatorin [9‐(3‐methyl‐2‐enoxy)‐7H‐furan[3,2g]chromene‐7‐one] (molecular weight 270.28 g/mol) is a linear furocoumarin compound. It is isolated from *Angelica archangelica*. and *Peucedanum praeruptorum*., that are components of traditional Chinese medicine. Being a plant extract, imperatorin is widely available and inexpensive and has antioxidative and anti‐apoptotic effects.^20^, ^21^, ^22^ QIN Lu‐Ping et al showed the osteogenic effect of imperatorin on human osteosarcoma cells to treat osteoporosis.^23^ The research by Xia Lv et al suggested the beneficial role of imperatorin in increasing growth of MC3T3‐E1 cells.^24^ However, the underlying mechanism and specific effect of imperatorin on bone metabolism have not yet been fully elucidated.

## 2 MATERIALS AND METHODS

Imperatorin was obtained from Sigma. The primary antibodies of COL1A1, RUNX2, Osteocalcin (OCN), BMP2, β‐catenin, GSK3β and p‐GSK3β (Ser9) were purchased from Abcam. The NFATC1, Histone H2A.X, phospho‐AKT (Ser473), and AKT antibodies were purchased from Santa Cruz Biotechnology. The c‐Fos, TRAP and phospho‐β‐catenin (Ser552) were purchased from Cell Signaling Technology. Foetal bovine serum (FBS), Minimum Essential Medium‐Alpha Modification (α‐MEM) and penicillin/streptomycin were purchased from Gibco BRL. All other chemicals were of analytical grade complying with the cell culture standards.

### 2.1 Experimental animals and treatment

In this study, we explored whether the in vitro effect of imperatorin on bone remodelling could be confirmed in vivo. Thirty 3‐month‐old SD male rats were procured from Shanghai Laboratory Animal Center (Shanghai, China). A group of five rats were kept in a cage at 12 hours/d light duration, 25°C, ventilated and dry. Access to tap water was ad libitum and standard laboratory diet was provided by Provimi Kliba AG (Kaiseraugst, Switzerland) containing 2.5% casein, 0.8% phosphorus, 1% calcium, 70%‐80% carbohydrates and 5% fat. All surgical interventions, treatments and post‐operative animal care procedures were performed as per the Animal Care and Use Committee, Wenzhou Medical University. After 1 week of adaptation, ten rats were subject to sham operation and the remaining rats underwent bilateral ovariectomy (OVX) as per protocol previously reported.^25^ After 12 weeks, imperatorin (Solarbio) or normal saline (NS) was administered. Further, the rats were divided into three groups (N = 10/group): SHAM group which underwent sham operation received NS (50 mL/kg/d) by gavage; OVX group which underwent bilateral ovariectomy also received (50 mL/kg/d) NS by gavage; whereas, the other ovariectomy group, IMP received imperatorin (20 mg/kg/d) by gavage. Gavage feeding was done once/day for 12 weeks.

### 2.2 Osteoblastic cell culture and assays

To evaluate the effect of imperatorin on osteogenic differentiation, BMSCs were chosen. BMSC is a kind of multifunctional stem cells widely used in bone tissue engineering. Some studies have found that it has the application value in craniomaxillofacial bone regeneration.^26^

Briefly, BMSCs were collected by flushing bone marrow of the 2‐week‐old SD rats’ femurs and tibiae with α‐MEM (*v/v*), 10% FBS, 100 U/mL penicillin and 100 μg/mL streptomycin. Cells were cultured at 37°C, 5% CO_2_ for 48 hours to allow initial cell adherence to culture flasks. Upon 80%‐90% confluence, cells were passaged, purified, seeded into 6‐well plates (5 × 10^5^ cells/well) and grown in complete α‐MEM until confluent. Osteoblast differentiation was induced by treating cells with medium containing 50 mmol/L ascorbic acid, 10 mmol/L β‐glycerophosphate, 10 nmol/L dexamethasone and 20 μmol/L ascorbic acid (Sigma), and the medium was replenished every 2 days. Alkaline Phosphatase (ALP) activity was measured using assay Kit (Beyotime) on the 7th day of differentiation. On the 21st day of differentiation, Alizarin Red S (ARS; Solarbio) staining was done to measure the degree of calcium deposition in the cells.

### 2.3 In vitro osteoclastogenesis assay

The femurs and tibiae of 2‐week‐old Sprague Dawley (SD) rats were flushed with α‐MEM containing 10% FBS, penicillin (100 U/mL) and streptomycin (100 mg/mL) to obtain bone marrow cells (BMCs). To obtain bone marrow‐derived monocytes (BMMS), BMCs were inoculated in α‐MEM supplemented with 10% FBS and 30 ng/mL macrophage colony‐stimulating factor (M‐CSF). After 4 days, unattached cells were removed, and adherent cells acted as the osteoclast‐precursor BMMS that were subsequently seeded in 96‐well plates (6000 cells/well) and cultured for 5 days with/without imperatorin in complete medium containing M‐CSF (30 ng/mL) and RANKL (50 ng/mL). The culture medium was changed twice a day and the cells were fixed with 4% paraformaldehyde for 10 minutes, infiltrated with 0.1% Triton X‐100 and then stained with tartrate‐resistant acid phosphatase (TRAP; Sigma). The TRAP‐positive multinucleated cells were enumerated, and cells with ≥3 nuclei were categories as osteoclasts.

### 2.4 Imperatorin cytotoxicity assay

The effect of imperatorin on the activity of BMMS or BMSC in bone marrow stromal cells was determined by cell counting kit (CCK‐8; MedChemExpress). The cells were inoculated in 96‐well plate (5000 cells/well) and cultured for 5 days (BMMS) or 7 days (BMSC) in the presence of different concentrations of imperatorin. CCK8 working fluid with a total volume of 10% of the total liquid volume is added to each hole and incubated for 4 hours. The optical density (OD) at the wavelength of 570 nm was determined by enzyme‐linked immunosorbent assay (ELISA; Multiskan Go, Thermo).

### 2.5 Quantitative real‐time RT‐PCR

Quantitative real‐time reverse transcription polymerase chain reaction (RT‐PCR) was used to measure the osteogenic expression of mRNA in BMSCs induced by osteoblasts under different treatments. The PrimeScript RT reagent kit (Takara Bio) was used to obtain total cell RNA and cDNA content as per manufacturer's instructions. The primers used to detect the genes of interest were as follows: Runx2, forward 5′‐CTTCCAGACCAGCAGCACTCCAT‐3′ and reverse 5′‐CCATCAGCGTCAACACCATCATT‐3′; OCN, forward 5′‐GCCATAGATGCGCTTGTAG‐3′ and reverse 5′‐TAAGGTGGTGAATAGACTCCG‐3′; Bmp2, forward 5′‐ATCCACTCCACAAACGAGAAAAGC‐3′ and reverse 5′‐CCCACATCACTGAAGTCCACATACA‐3′.

Wnt3a, forward 5′‐TGCAGGAACTACGTGGAGATCA and reverse 5′‐ GGCATGGACAAAGGCTGACTC; GAPDH, forward 5′‐TCTCTGCTCCTCCCTGTTC‐3′ and reverse 5′‐ACACCGACCTTCACCATCT‐3′. The amplification parameters included an initial denaturation at 95°C for 5 minutes, then denaturation at 95°C for 1 minute, annealing at 60°C for 30 seconds and extension at 72°C for 1 minute, repeating 40 cycles. Melting point analysis was used to confirm the specificity of the SYBR green assay. Data were converted to mRNA expression fold using ddCt comparative threshold cycle method.

### 2.6 Western blot assay

Total cell protein was obtained using radioimmunoprecipitation assay buffer (RIPA) lysis buffer (Beyotime). Nuclear proteins were obtained using NE‐PER Nuclear and Cytoplasmic Extraction Reagents (Thermo) according to the manufacturer's instructions. Protein concentration was determined using the BCA Protein Assay Kit (Beyotime). Protein (20 mg) was subjected to SDS‐PAGE using a 10%‐15% concentration gradient gel and transferred to the polyvinylidene difluoride (PVDF) membrane (Millipore). The PVDF membrane was blocked with 5% skim milk diluted with 0.1% Tween‐20 in tris‐buffered saline (TBST) for 2 hours. The primary antibody was incubated for 12 hours at 4°C and washed three times with TBST for 5 minutes each. Secondary antibody was incubated for 4 hours at room temperature. The final step was to quantify the protein strength on the membrane using Image Lab 3.0 software (Bio‐Rad).

### 2.7 Immunofluorescence

Osteogenesis was induced in BMSCs with treatment of imperatorin at different concentrations. After 7 days, cells were fixed with 4% paraformaldehyde solution and permeabilized with 0.2% Triton X‐100 for 15 minutes and non‐specific binding was blocked with 10% goat serum solution. Cells were then incubated overnight at 4°C with the primary antibodies: COL1A1, RUNX2, OCN, BMP2 and β‐catenin, after which they were washed with PBS and incubated for 1 hour at room temperature with an appropriate Alexa fluorescent‐conjugated antibody (Molecular Probes, Life Technologies) in 1:400 dilution. Finally, the plate was stained with DAPI Fluoromount‐G and anti‐fluorescence quenched with DAPI Fluoromount‐G^™^ (YEASEN). Stained sections were observed under fluorescence microscope (Olympus BX53; Olympus Corporation).

### 2.8 Specimen collection

After 12 weeks, all rats were euthanized after anaesthesia, then the bilateral femurs were removed and muscles were stripped. Soft tissue was fixed with 4% paraformaldehyde.

### 2.9 Microtomography analysis

Micro‐CT imaging system (Micro‐CT CT50, Scanco Medical) was used to evaluate microstructure of the distal femur. The volume of interest (VOI) included the trabecular compartment from 2.0 microstructure mm below the highest point of the growth plate to distal 100 slices. Three‐dimensional images and related parameters such as the per cent bone volume (BV/TV), the mean trabecular thickness (Tb.Th), the mean trabecular number (Tb.N), the mean trabecular separation (Tb.Sp) and the mean connective density (Conn.D) were acquired for qualitative and quantitative analysis of VOI zone.

### 2.10 Histomorphology analysis

Next, the samples were decalcified in 10% ethylenediaminetetraacetic acid (EDTA), changed twice/week for 3 weeks and then dehydrated through graded ethanol series (70%‐100%). Paraffin embedding was done subsequently with the long axis parallel to the base plane. Longitudinal serial sections of 4 mm thickness were cut and mounted on poly‐lysine–coated microscope slides and then subjected to H&E staining. The staining was performed according to manufacturer's protocol and examined under a microscopic light (Olympus DP71 microscope, Olympus Co.).

### 2.11 Immunostaining

To detect the levels of RUNX2 and TRAP, femurs were fixed in 10% neutral‐buffered formalin solution. After decalcified in 10% EDTA, tissues were dehydrated through a graded alcohol series, cleared in xylene and embedded in paraffin. Tissue sections of 6 mm thickness were mounted on glass slides and subjected to immunohistochemistry (IHC) staining according to manufacturer's protocol with the specific kit (Vector Laboratories). Positively stained osteoblasts and osteoclasts were enumerated using image analysis software.

## 3 RESULTS

### 3.1 Imperatorin stimulates osteoblast differentiation and mRNA expression of osteoblastic gene markers

**Figure 1:**
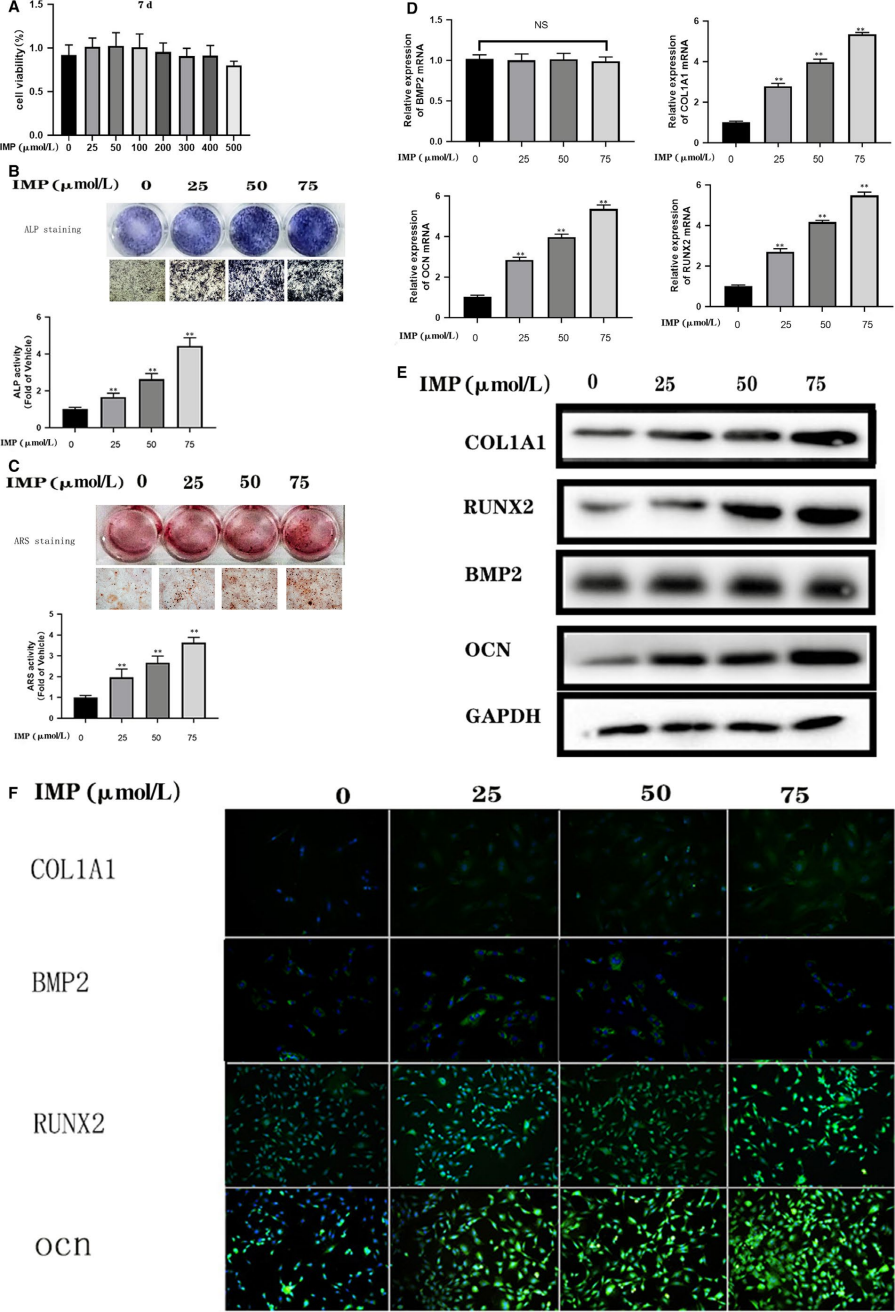
Imperatorin promotes osteogenic differentiation of BMSCs in vitro. A, Effects of different concentrations of imperatorin on the growth of BMSCs, during osteogenesis on the 7th day, as measured by CCK8 method. Osteogenic induction of BMSCs in vitro, with different concentrations of imperatorin intervention from the first day of induction; B, ALP staining on the 7th day; C, and mineralization degree by the ARS staining on the 21st day; D, the mRNA expression levels of COL1A1, RUNX2, OCN and BMP2 were measured by real‐time RT‐PCR; E, the protein expression levels of the above indicators were analysed by Western blot; F, representative immunofluorescence images of the above indicators, counterstained with DAPI. Data were expressed as mean ± SD, n = 5; *P < .05, **P < .01 vs IMP (50 umol/L) group

As shown in Figure 1A, IMP with a concentration below 400 μmol/L has no cytotoxicity to BMSCs. To evaluate the effect of imperatorin on osteogenic differentiation of BMSCs, staining was done and activity of the early osteogenic differentiation marker, ALP was examined on 7th of culture. As shown in Figure 1B, imperatorin significantly *P* < .05 increased the expression and activity of ALP. Osteoblasts are responsible for the mineralization of the extracellular matrix (ECM), the final step in osteoblast differentiation. To investigate the role of imperatorin in inducing mineralization, osteoblasts were stained with ARS after 21 days of culture. In differentiated osteoblasts without imperatorin, ECM calcium deposition was less; whereas, substantial ARS staining was observed in differentiated cells treated with imperatorin. The mineralization increased with increase in the concentration of imperatorin (Figure 1C). The results indicated that imperatorin enhanced the initial differentiation of osteoblasts by increasing ALP activity and late‐stage differentiation by increasing mineralization. Next, we examined the expression of genes involved in osteoblast differentiation. The expression levels of *RUNX 2, OCN, COL1A1* in the imperatorin treatment group were significantly higher than that in the control group (Figure 1D). However, no difference was observed in the expression levels of BMP2, indicating that the enhancing effect of imperatorin on osteogenesis was not through the BMP2/SMAD pathway. Further, our results of Western blot analysis were in agreement with these results.

### 3.2 Imperatorin promotes osteogenesis via AKT/ GSK3β/ β‐catenin axis

**Figure 2:**
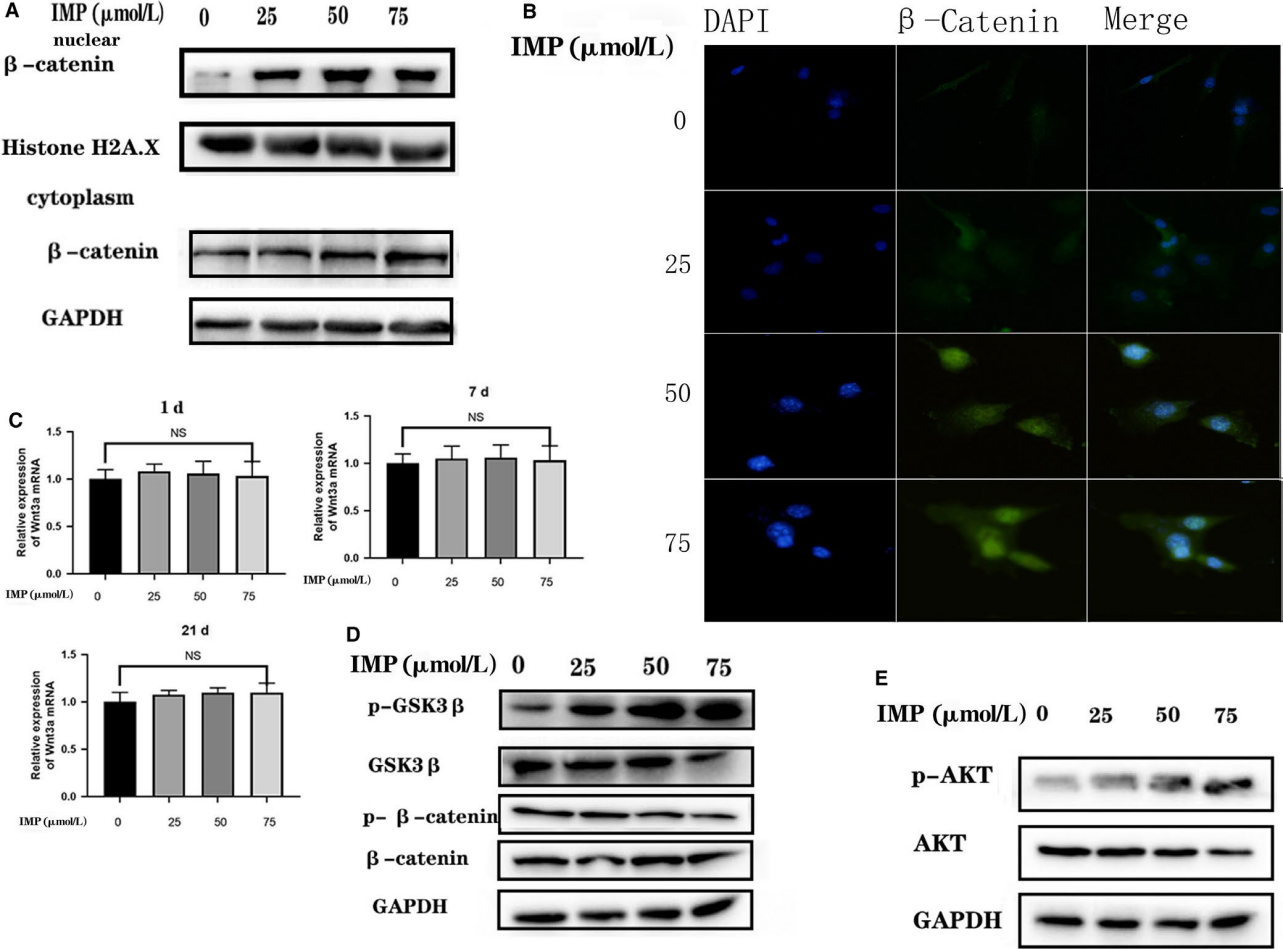
Imperatorin promotes osteogenesis via AKT/GSK3β/β‐catenin axis. A, Nuclear and cytoplasmic proteins were isolated, and the amount of β‐catenin was detected by Western blot; B, representative immunocytochemistry images showing imperatorin increased the β‐catenin in the nucleus; C, on the 1st, 7th and 21st day during the osteogenic differentiation of BMSCs, RNA was extracted to detect the transcription level of Wnt3a; D, E, Western blot examination showed that the expression of phospho‐AKT (Ser473), AKT, phospho‐β‐catenin (Ser552) and β‐catenin, p‐GSK3β (Ser9) and GSK3β changed after the intervention of imperatorin on osteogenic differentiation of BMSCs

**Figure 3:**
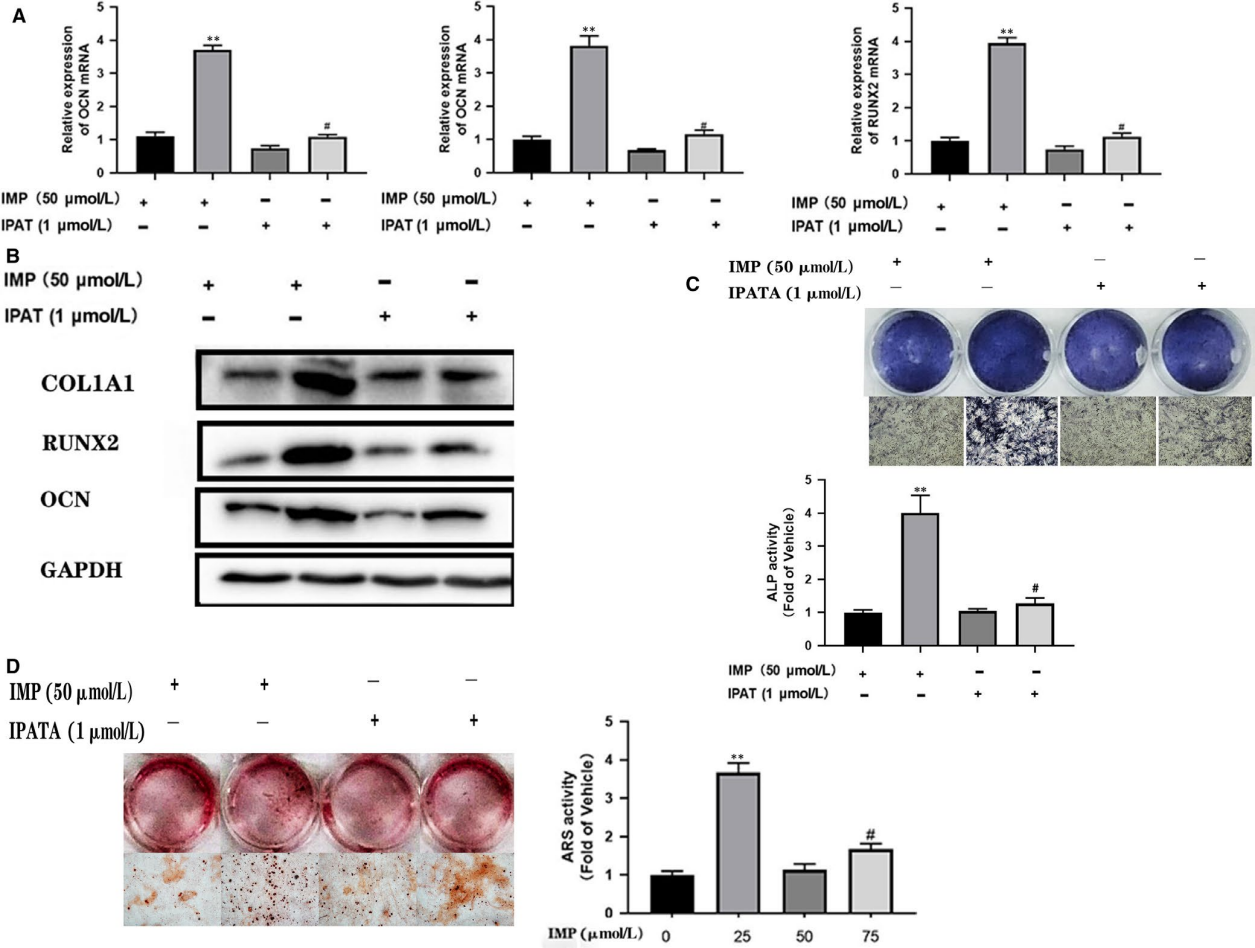
Ipatasertib inhibits the effect of imperatorin on osteogenic differentiation. The osteogenic differentiation was induced in BMSCs in vitro and divided into four groups according to the intervention of imperatorin and ipatasertib or not. On the 7th day of differentiation, A, the transcription levels of COL1A1, OCN and RUNX2 were detected by qRT‐PCR; B, detection of the expression of COL1A1, OCN and RUNX2 proteins by Western blot; C, ALP staining; D, On the 21st day, the degree of mineralization was detected by ARS staining

The results of Western blot and immunofluorescence suggested that imperatorin increases the amount of β‐catenin in the nucleus (Figure 2A,B). The Wnt/β‐catenin is a classical pathway that influences the osteogenic differentiation. The β‐catenin enters the nucleus to promote transcription of downstream osteogenic‐related genes, of which RUNX2 is the major target.^27^ The rt‐PCR results indicated that imperatorin did not directly increase the Wnt3a expression in BMSCs during osteogenic differentiation (Figure 2C). However, during osteogenic induction, the phosphorylation of GSK3β was promoted and β‐catenin was inhibited (Figure 2D). Studies have shown that AKT acts as an upstream signal to regulate the AKT/GSK3β/β‐catenin signal axis.^28^, ^29^, ^30^ Our results suggest that imperatorin promotes phosphorylation of AKT (Figure 2E). To verify that AKT plays an essential role in imperatorin promoting osteogenesis, we selected ipatasertib (IPATA; Selleck), a novel, highly selective, pan‐AKT inhibitor.^31^, ^32^, ^33^, ^34^ After pre‐treatment with IPATA (1 µmol/L) for 24 hours, compared with the control group, the IMP treatment group showed more significant osteogenic *P *< .05 differentiation. However, the osteogenic differentiation was not as apparent as in seen the group without IPATA intervention (Figure 3). This result indicates that the effect of imperatorin on BMSCs promoting osteogenic differentiation was inhibited by IPATA, suggesting that imperatorin acts by stimulating AKT/GSK3β/β‐catenin pathways.

### 3.3 Imperatorin inhibits RANKL‐induced osteoclast differentiation

**Figure 4:**
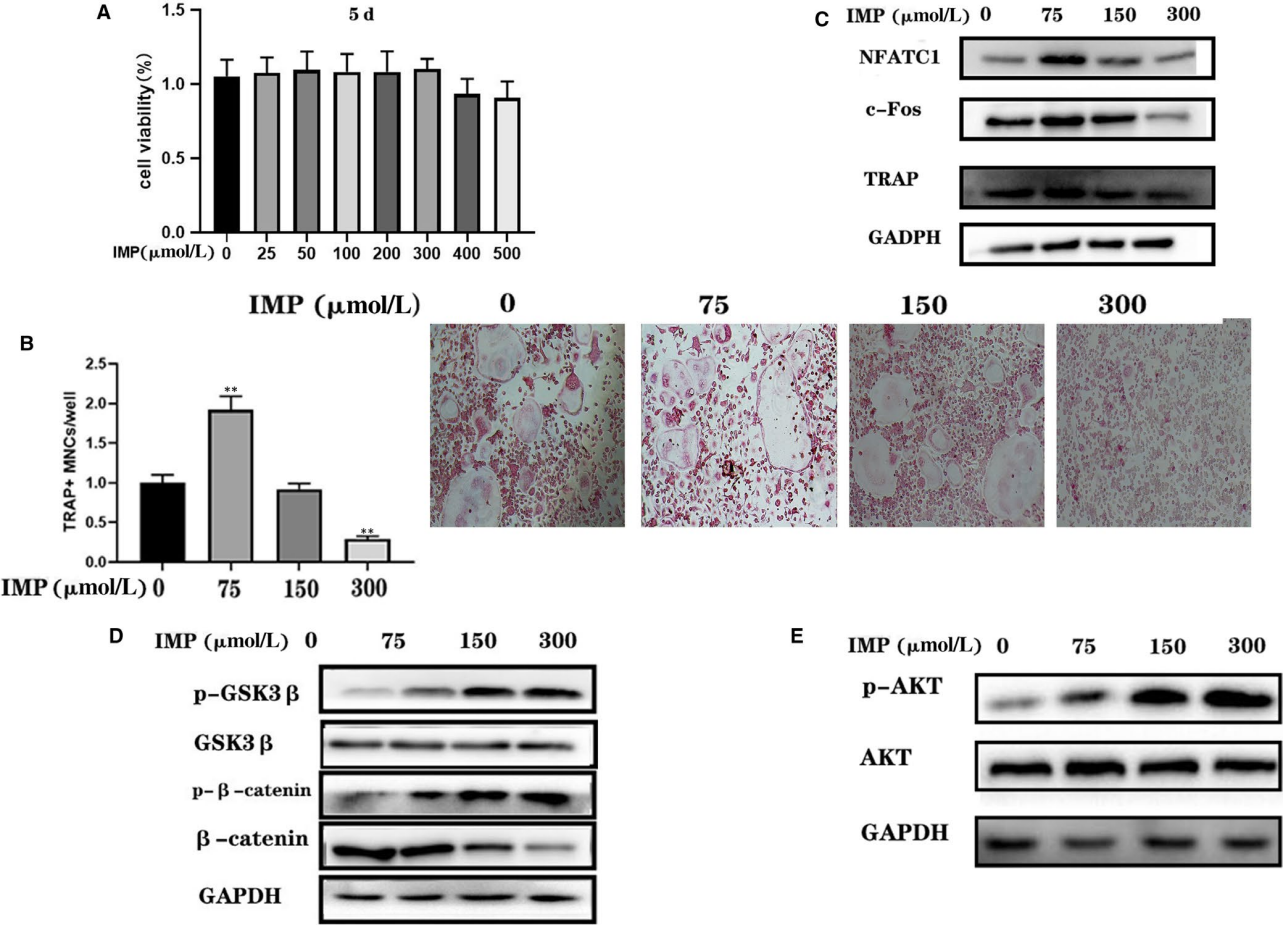
Effect of imperatorin on osteoclast differentiation of BMMS induced by RANKL. A, The CCK‐8 experiment showed the effect of different concentrations of imperatorin on the growth of BMMS on the 5th day. The BMMS was induced to differentiate into osteoclasts by RANKL with 50 ng/mL concentration in vitro; B, TRAP staining was performed on the 5th day after intervention with different concentrations of imperatorin; C, detection of the expression of NFATC1, c‐Fos and TRAP by Western blot; D, after BMMS was stimulated with imperatorin for 30 min, the protein was extracted and the expression of p‐GSK3β(Ser9), GSK3β and p‐β‐catenin(Ser552) and β‐catenin was analysed; E, the expression of phospho‐AKT(Ser473) and AKT was analysed. Data were expressed as mean ± SD, n = 5; *P < .05, **P < .01 vs IMP (300 μmol/L)

Next, we studied the effect of imperatorin on osteoclast differentiation. We pre‐stimulated BMMS with imperatorin, and then induced osteoclast differentiation with RANKL. In the presence of M‐CSF, RANKL can induce BMMS to differentiate into osteoclasts, and the mature osteoclasts were TRAP‐positive multinucleated cells. The TRAP staining showed that imperatorin promoted osteoclast differentiation at low concentration (25 µmol/L), but inhibited at high concentration (75 µmol/L; Figure 4B). The results of Western blot showed that the expression of NFATC1, c‐Fos and TRAP increased at low concentration of imperatorin and decreased at high concentration of imperatorin (Figure 4C). Imperatorin activated β‐catenin in BMMS through AKT/GSK3β pathway (Figure 4D,E).

**Figure 5:**
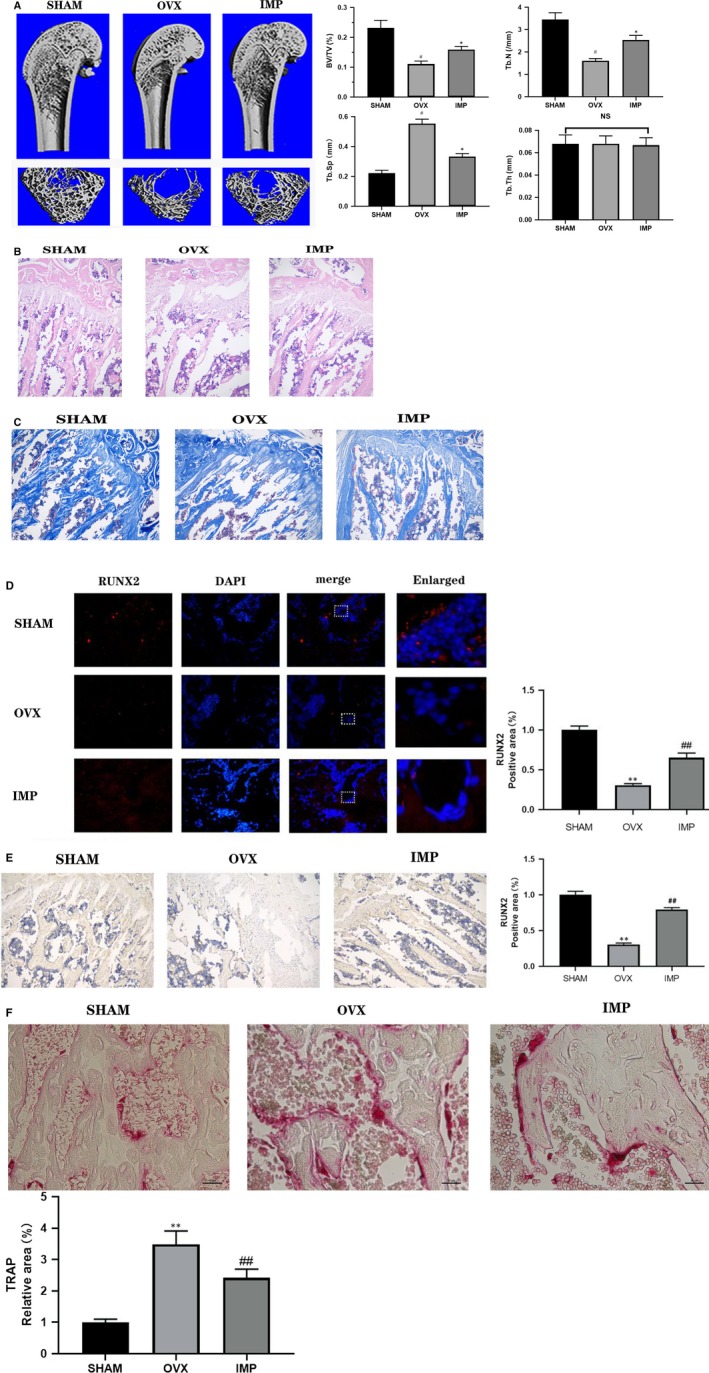
Effect of imperatorin on osteoporosis caused by oestrogen deficiency. A, After euthanasia, the femurs of the rats were removed and the X‐ray images of the longitudinal and transverse sections of the distal femurs were taken with a miniature CT device. Quantitative analysis results of BV/TV, Tb.N, Tb.Sp and Tb.Th were divided into three groups; B, H&E staining; C, Masson's staining of metaphyseal tissue sections of thigh (D) fluorescence and quantitative analysis, (E) histochemical and quantitative analysis, (F) quantitative TRAP staining and analysis of RUNX2 tissue in metaphyseal tissue section of femoral shaft. Data were expressed as mean ± SD, **P < .01 vs SHAM, ##*P* < .01 vs OVX

To confirm the role of imperatorin in promoting bone formation, we performed in vivo experiments in rats as aforementioned. One rat died each group that is OVX and imperatorin; whereas, all the rats from the SHAM group survived to collect femoral specimens. The bone volume of OVX rats was significantly lower than that of the SHAM group; however, imperatorin reversed this condition in OVX rats. According to the results of quantitative analysis, BV/TV, Conn.D, Tb.N, Tb.Th in IMP group were closer to SHAM group, than OVX group (Figure 5A). The H&E and Masson's staining revealed that imperatorin increased the number of trabecular bone, reduced the trabecular space and improved the structural arrangement of the bone in OVX rats (Figure 5B). Imperatorin enhanced the activity of osteoblasts and decreased the activity of osteoclasts in the metaphyseal tissue of OVX rats.

To further verify the effects of imperatorin on osteoblasts and osteoclasts in vivo, we used the aforementioned rat metaphyseal tissue sections for RUNX2 immunohistochemical, immunofluorescence and TRAP staining. As shown in the Figure 5D,E, the osteogenic activity of rat femoral metaphysis decreased after ovariectomy, but partially recovered after imperatorin intervention. The results of TRAP staining suggested that in oestrogen deficiency, the osteoclast activity of the metaphysis of the rat was enhanced, and imperatorin could partially prevent this phenomenon (Figure 5F).

## 4 DISCUSSION

At present, bisphosphonates, raloxifene and oestrogens are the drugs used for the treatment of bone diseases. Although they all have certain curative effects, they also have different limitations including thromboembolism and oesophageal irritation.^5^, ^6^, ^7^ The primary goal of our treatment was to restore the normal metabolism of the bones, and balance the osteogenesis and osteolysis, that is, enhance the osteogenic function and reduce the bone‐weakening due to pathological condition of osteoporosis. Therefore, we intended to find a potential drug for its treatment from natural compounds. In this study, we found that imperatorin increases the bone mineral density by promoting osteogenesis and inhibiting osteoclast formation both, in vivo and in vitro.

Imperatorin [9‐(3‐methyl‐2‐enoxy)‐7H‐furan [3, 2 g] chromene‐7‐one] (molecular weight 270.28) is a linear furan coumarin compound, which is A.archangelica and White Flower Peucedanum (Peucedanum precantorum L.) components of traditional Chinese medicine. As a plant extract, imperatorin is widely available and inexpensive. Many reports have suggested that imperatorin has antioxidant and anti‐apoptotic effects.^17^, ^18^, ^19^ QIN Lu‐Ping and his team found that imperatorin showed osteogenesis on Saos‐2 cells and has the potential to treat osteoporosis.^20^ But the specific mechanism is not understood. Thus, we carried out the experiment to observe the influence of imperatorin on the osteoblast and osteoclast using osteoporotic rats. Our results suggested that imperatorin promotes the expression of ALP, implying that it plays a positive role in early stages of osteogenesis. Whereas, the results of ARS staining suggested that imperatorin promoted the mineralization of osteoblasts, indicating that it also plays a role in promoting the late osteogenic process. The outcomes of qRT‐PCR and Western blot supported that imperatorin promotes the maturation and differentiation of osteoblast by enhancing the expression of RUNX2, OCN and COL1A1. The RUNX2 is an important indicator of the degree of early stage of osteogenic differentiation, and also a primary regulatory factor of osteocalcin, playing a key role in osteogenesis.^33^, ^34^, ^35^ Whereas, COL1A1 is a component of Col1, the most abundant protein in the bone matrix, and is directly involved in the mineralization and maturation of osteoblasts. The results of immunofluorescence also supported the conclusions. Further, we found that imperatorin can stimulate the classical osteogenic pathway, Wnt/β‐catenin. Whereas, the results of Western blot showed that imperatorin‐activated AKT resulted in the inactivation of GSK3β, thus inhibiting the phosphorylation of β‐catenin and eventually leading β‐catenin to enter the nucleus. Interestingly, the ipatasertib could significantly inhibit this osteogenic effect of imperatorin. These results suggest that imperatorin promotes osteogenesis through the AKT/GSK3β/β catenin pathway.

In addition, IMP plays a role in inhibiting osteoclast formation induced by RANKL. Using human osteoclast progenitor cells isolated from patients with multiple myeloma as the experimental object, John D. Shaughnessy Jr found that the differentiation of human osteoclasts was inhibited by Wnt/β‐catenin pathway.^36^ Wnt cascade is initiated by the binding of Wnts to LRP/Fzd receptor complex, leading to the recruitment, phosphorylation and inactivation of GSK3β. This leads to the activation of β‐catenin and the accumulation of β‐catenin in the nucleus 37, 38, 39, 40 However, our results suggest that although imperatorin can inhibit the differentiation of osteoclasts and the expression of NFATC1 and TRAP at high concentration (75 µmol/L), it can promote the osteoclast differentiation and expression of these genes at low concentration (25 µmol/L). Osteoclast differentiation is a process involving cell proliferation, commitment, fusion and activation.^41^ The activation of β‐catenin plays an important role in the proliferation of osteoclast progenitor cells; however, it plays an inhibitory role in its differentiation to osteoclasts.^42^ This study found that the overexpression of β‐catenin can inhibit the differentiation of osteoclasts in vitro, supporting the earlier claims.^43^ However, Jang HD et al found that GSK3β could inhibit osteoclast differentiation. The promotion of osteoclast differentiation and overexpression of GSK3β after silencing of GSK3β could inhibit the osteoclast differentiation induced by RANKL.^44^ The inactivation (phosphorylation) of GSK3β is the intermediate product after the activation of Wnt/β‐catenin pathway.^37^, ^38^ Further, GSK3β plays an important role in many pathways, including, growth factors, hedgehogs, G‐protein coupling ligands, cytokines and Wnt.^45^, ^46^ It is also a key downstream factor of AKT and plays an essential role in osteoclast differentiation. Our results show that low concentration of imperatorin activates the AKT/GSK3β/NFATC1 axis in BMMS cells. We speculate that intervention with low concentration of imperatorin promotes the osteoclast differentiation of BMMS by activation of AKT/GSK3β/NFTAC1 pathway.^47^, ^48^ When the concentration of imperatorin was high, a large number of activated β‐catenin inhibited the osteoclast differentiation. Neither the inhibitory effect of Wnt/β‐catenin pathway on osteoclast nor the role of AKT/GSK3β/NFTAC1 pathway in promoting osteoclast influenced c‐Fos. The c‐Fos is an upstream transcription factor of NFATC1 which plays an important role in RANKL‐induced osteoclast differentiation in conjunction with intracellular calcium concussion.^49^

Further, we removed the bilateral ovaries to establish a rat model of osteoporosis, an animal model of osteoporosis reflecting postmenopausal women as recommended by FDA.^50^ After 12 weeks of administration of imperatorin, we collected distal femoral specimens from rats. The results of H&E and Masson's staining showed that imperatorin significantly promoted the formation of bone trabeculae. Additionally, the microscopic CT scans showed the 3D results and detailed parameters. Compared with OVX group, BV/TV and Tb. N in imperatorin group were closer to those in SHAM group. Gradually, the Tb.Sp decreased and the bone structure improved.

In conclusion, imperatorin modulates bone remodelling by stimulating osteoblast function and inhibiting osteoclast differentiation. This suggests that imperatorin could be used as a safe and an effective dual‐action therapeutic agent against osteoporosis that could promote bone growth and inhibit resorption. Although IMP has been shown to be effective in the treatment of osteoporosis, systemic administration often requires the delivery of hyperphysiological factors to achieve efficacy, leading to the risk of harmful side effects.^51^ The specific peptides, aptamers and phosphate‐rich compounds which can be formed in the bone microenvironment can be used as targeting carriers to enhance the bone regeneration of non‐targeted drugs and reduce the effect of extra‐target tissue.^52^

## CONFLICT OF INTEREST

All authors have no conflicts of interest.

## AUTHOR CONTRIBUTIONS

All the listed authors made substantial contributions to the study. Lei Yang and De‐Yi Yan, Jiahao Tang, Liang Chen, Bingzhang Wang participated in the experimental design and contributed reagents, materials and analytical tools. Sheji Weng, Zhongjie Xie, Zong‐Yi Wu and Zijian Shen, Bingli Bai were also involved in the experiment. Lei Yang and De‐Yi Yan wrote the manuscript. De‐Yi Yan, Liang Chen, Bingzhang Wang and Sheji Weng, Zhongjie Xie are involved in data analysis. All the authors read and approved the final manuscript. All the data shown in the figure are from the above authors' experiments and can be used.

## ETHICAL APPROVAL

The study was approved by the Animal Experimentation Ethics Committee of Second Affiliated Hospital, Wenzhou Medical University.

## Data Availability

The data used to support the findings of this study are available from the corresponding author upon request.
